# Design and Implementation of an Educational Program in Advanced Airway Management for Anesthesiology Residents

**DOI:** 10.1155/2012/737151

**Published:** 2012-02-28

**Authors:** Zana Borovcanin, Janine R. Shapiro

**Affiliations:** ^1^Advanced Airway Management Educational Program, Department of Anesthesiology, University of Rochester School of Medicine and Dentistry, 601 Elmwood Avenue, P.O. Box 604, Rochester, NY 14642, USA; ^2^Department of Anesthesiology, University of Rochester School of Medicine and Dentistry, 601 Elmwood Avenue, P.O. Box 604, Rochester, NY 14642, USA

## Abstract

Education and training in advanced airway management as part of an anesthesiology residency program is necessary to help residents attain the status of expert in difficult airway management. The Accreditation Council for Graduate Medical Education (ACGME) emphasizes that residents in anesthesiology must obtain significant experience with a broad spectrum of airway management techniques. However, there is no specific number required as a minimum clinical experience that should be obtained in order to ensure competency. We have developed a curriculum for a new Advanced Airway Techniques rotation. This rotation is supplemented with a hands-on Difficult Airway Workshop. We describe here this comprehensive advanced airway management educational program at our institution. Future studies will focus on determining if education in advanced airway management results in a decrease in airway related morbidity and mortality and overall better patients' outcome during difficult airway management.

## 1. Introduction

Anesthesiologists are recognized as experts in difficult or failed airway management. However, anesthesiology residents are not exposed frequently enough to a difficult or failed airway during the course of their three years of clinical training in order to attain “expert” status in difficult or failed airway management. The *American Society of Anesthesiologist *(ASA)* Practice Guidelines for Management of Difficult Airway *suggests that when conventional intubation techniques fail after three attempts, advanced airway management devices or techniques should be utilized and immediately available [1]. A comprehensive advanced airway management educational program as a part of an anesthesiology residency program is necessary to help anesthesiology residents earn the status of expert in difficult airway management [2–4] and be able to use successfully advanced airway management devices or techniques when faced with a difficult airway. Three years ago we implemented an Advanced Airway Techniques (AAT) rotation as a new two- to four-week rotation for residents in anesthesiology during their third year of clinical anesthesia training (CA-3 year). This educational activity is supplemented with a Difficult Airway Workshop, a semiannual educational activity. Before initiation of this rotation, education in advanced airway management was sporadic at our institution consisting of occasional individual teaching as difficult airways arose. With implementation of this rotation, a formal advanced airway management program was instituted. We describe below details of this educational program.

## 2. Comprehensive Advanced Airway Management Educational Program

### 2.1. Educational Goals and Planning

The goals of the comprehensive advanced airway management educational program are to enable residents to obtain significant experience with a broad spectrum of advanced airway management techniques and devices, learn to appropriately apply the ASA Difficult Airway Algorithm, and develop an understanding of the critical decision points in the course of the difficult or failed airway management. In order to successfully implement such a program, it is necessary to develop a specific curriculum, have excellent airway equipment always available, and have a core group of faculty with expertise in advanced airway management. The proposed curriculum detailed below was accepted by the program director and the resident education committee. Our department has a core group of faculty with expertise in advanced airway management. Standardized difficult airway carts, uniformly stocked and set up, are utilized during the education and training in advanced airway management techniques.

### 2.2. Advanced Airway Techniques (AAT) Rotation

#### 2.2.1. Curriculum

The AAT rotation is an elective rotation offered to the CA-3 resident for a duration of a minimum of two weeks and a maximum of four-weeks. Resident responsibilities are to read the goals and objectives of the rotation, become familiar with the classic and current literature on advanced airway management, and complete the log sheets daily during the rotation. The patient's medical record number, the success and timing of the technique, and the supervising faculty name are written on the log sheet. The educational material for the AAT rotation is posted on Blackboard, is accessible to all residents, and is discussed by the faculty with the residents during the rotation. The educational material includes the introductory article *ASA Practice Guidelines for Management of Difficult Airway *published in 2003 [1]. The additional material is grouped according to the advanced airway devices and techniques for the difficult airway management [5–39]. Every advanced airway management device or technique used during the rotation is discussed with resident with regards to description, instruction on insertion technique, and current clinical use. Adequate supervision of the resident by the faculty with expertise in advanced airway management is important to master the techniques. Selection of the patients is also important. ASA l or ll patients with a class l or ll airway undergoing elective surgery under general anesthesia are ideal. Patients with a known or suspected difficult airway, as well as patients who are candidates for awake flexible fiberoptic intubation, are assigned to the resident on the AAT rotation.

Education and training in flexible fiberoptic laryngoscopy and intubation emphasizes the approach and skills needed for awake flexible fiberoptic intubation (FFI). Troubleshooting of FFI is taught. Residents become competent in the mechanical manipulation of the fiberoptic bronchoscope, learn to identify normal pharyngeal and laryngeal anatomy, and confirm the proper placement of the endotracheal tube in the trachea. FFI is mostly performed on asleep paralyzed patients. The average number of flexible fiberoptic intubations performed per resident is ten to twenty, and depends of the duration of the rotation. Johnson and Roberts suggested that an acceptable level of technical expertise in fiberoptic intubation can be obtained by the tenth intubation [7]. In this study, greater than a 95% success rate for the first attempt was achieved by performing ten elective asleep fiberoptic intubations by anesthesiology residents with no prior experience with fiberoptic intubation. This study demonstrated that ten fiberoptic intubations enabled residents to meet the learning objectives in the use of fiberoptic laryngoscopy and intubation. We have had a similar experience. There has a been a significant improvement in residents' skills during the rotation with a decrease in the time needed for a successful FFI from approximately three minutes down to thirty seconds. For awake intubation cases, the use of airway blocks and proper topical anesthetic techniques of the airway are taught. Literature describing the techniques for the airway blocks is posted for review [8]. FFI via supraglottic airway device using the Aintree intubation catheter is one of the techniques that our residents master during the AAT rotation which can be used successfully in the “cannot ventilate cannot intubate” situation [9, 10]. During the troubleshooting of FFI, video laryngoscopy is used to identify problems with the advancement of the endotracheal tube through the glottic opening. Video laryngoscopy is used as a teaching tool during the AAT rotation. Our residents become proficient with video laryngoscopy technique very early during their training. Several GlideScope video laryngoscopes are available and every difficult airway cart is supplied with a Mac video laryngoscope. The combined technique of FFI and video laryngoscopy is described by Doyle [11]. Educational material discussing video laryngoscopy techniques and devices is also available [12–20]. Possible complications associated with delivery and advancement of the endotracheal tube (ETT) with the GlideScope video laryngoscope, such as right palatopharyngeal arch perforation, is emphasized during the teaching of the proper video laryngoscopy technique [19]. Understanding of the mechanical and optical complexities of ETT insertion in video laryngoscopy is critical to facilitate rapid endotracheal intubation and to minimize unsuccessful attempts and injuries [20].

Supraglottic airway devices, the intubating laryngeal mask airway (LMA) and the ProSeal LMA are used during the rotation. Explanation of the devices and techniques is available for review [21, 22]. The lightwand, an illuminating device, is one of the techniques that our residents learn to master during the AAT rotation. A comprehensive review of the lighted stylet technique is available as an educational material [23]. Optically enhanced laryngoscopy is one of the new techniques used for advanced airway management. In our department, Airtraq is available in three sizes and can be used during rotation. Intubation with optical stylets, Levitan and Bonfils, is taught as one of the techniques for the advanced airway management. Literature discussing these devices and techniques is also posted for review [26–31]. Residents also become familiar with airway devices for endotracheal tube change (adult and pediatric Cook Airway Exchange Catheters), double-lumen tube exchange catheter and intubation catheter (Aintree intubation catheter) [32–34].

Invasive and surgical airway techniques for difficult airway management, such as retrograde wire intubation and cricothyrotomy are taught on the mannequin at our simulation center. Although rarely used in a daily routine, retrograde wire intubation is part of the airway curriculum. Surgical airway is practiced during the simulated code airway and residents become familiar with the Cook cricothyrotomy tray. Educational material for invasive airway is posted on Blackboard [35–37]; there is also a link to the Cook Medical videos explaining both procedures [38, 39].

#### 2.2.2. Equipment

**Figure 1:**
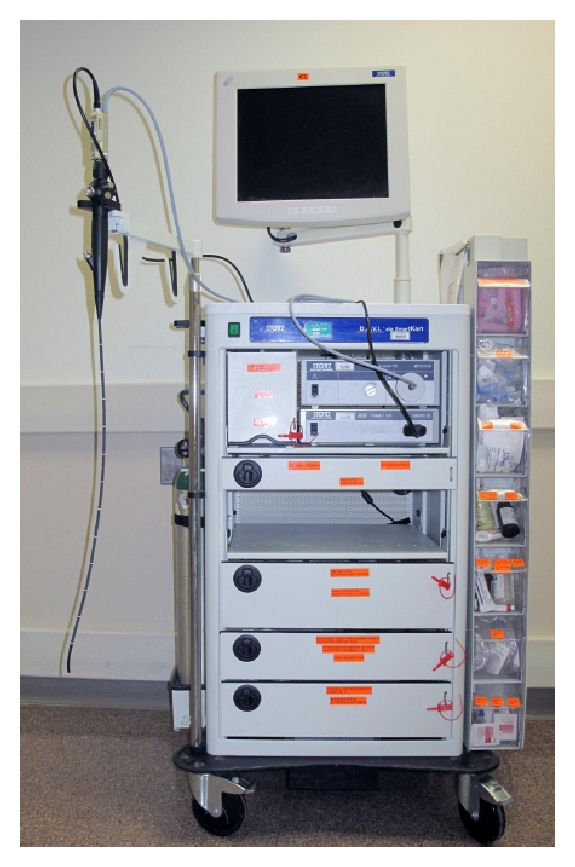


Standardized difficult airway carts (Figure 1) are used during the rotation. We currently have seven standardized difficult airway carts which are always available and located throughout our department. They are uniformly stocked and set up. Each difficult airway cart is equipped with a Difficult Airway Video Intubation (D.A.V.I.) system manufactured by Karl Storz Endoscope. Flexible fiberoptic bronchoscopes in adult (5.2 mm and 3.7 mm outer diameters) and pediatric (2.8 mm outer diameter) sizes are available on every difficult airway cart. The following advanced airway management devices are stocked in the drawers: laryngeal mask airway, intubating LMA, Combitube, lighted stylet, cricothyrotomy set, retrograde intubation set, emergency transtracheal airway catheter, jet-ventilation equipment, airway exchange catheters (pediatric and adult) and Aintree intubation catheter (see the appendix). Anesthesiology technicians are responsible for cleaning and restocking the equipment and supply after each use.

#### 2.2.3. Evaluation

An evaluation is performed through the E*Value system. The resident is required to maintain a log sheet with the number of procedures performed, and the success rate and time needed for each procedure. The rotation director provides each resident with formative and summative evaluation of performance. The resident is evaluated for the knowledge of airway anatomy and innervation and application of that knowledge to patient care during advanced airway management. If the resident has an opportunity to perform awake intubation during the rotation, competency in performing airway blocks (glossopharyngeal nerve block, superior laryngeal nerve block and transtracheal block) required for awake intubation is evaluated. Demonstration of competency with all used advanced airway management devices and techniques is also evaluated. During the code airway simulator practice, the resident is evaluated for appropriate application of the *ASA Difficult Airway Algorithm *and demonstration of the understanding of critical decision points in the course of the difficult airway management. During the simulation of crisis management, the resident should be able to declare the emergency, call for specific help, acknowledge and confirm information, issue orders and effectively communicate with the team members. The resident is evaluated for competency in surgical airway management on the mannequin using cricothyrotomy and retrograde wire intubation. The AAT rotation is evaluated by the residents through the “Survey of Educational Activities” conducted near the end of each academic year.

### 2.3. Difficult Airway Workshop

The Difficult Airway Workshop is a semiannual educational activity which supplements the Advanced Airway Techniques rotation as part of the comprehensive advanced airway management educational program in our department. After successful completion of the Difficult Airway Workshop, residents should be able to anticipate and manage the difficult airway, identify and manage the failed airway, perform an awake intubation in the predicted difficult airway, and perform a surgical airway in the failed airway (“cannot ventilate, cannot intubate” situation). It is an all-day teaching activity starting with didactic sessions in the morning followed by a rotation through the hands-on stations in the afternoon. The educational material for the Difficult Airway Workshop is also posted on Blackboard and is available and accessible to all residents. Original articles, review articles, book chapters, and videos are posted under the specific stations.

#### 2.3.1. Didactic Sessions

Series of clinical scenarios of difficult airway cases are presented and discussed during the didactic teaching focusing on developing critical decision-making processes in difficult airway management. Turning Point used during these problem based learning discussions facilitates interactive teaching. Evaluation of the airway for potential difficulty is emphasized during the analysis and explanation of the *ASA Difficult Airway Algorithm* [1]. Thorough history and physical examination of each patient for potential airway difficulty with predictors of anticipated difficult airway is stressed. Recognition and management of the child with a difficult airway is taught by a pediatric anesthesiologist with expertise in difficult airway management. An airway block session explains the anatomy and innervation of the nasal cavity, tongue, pharynx, larynx and trachea, and the different techniques for anesthesia of the airway necessary for awake video laryngoscopy or awake nasal or oral flexible fiberoptic intubation. Lung separation during difficult airway management and application of bronchial blockers is addressed by faculty with expertise in thoracic anesthesia and difficult airway management. Techniques, indications, and contraindications for retrograde wire intubation technique and emergency cricothyrotomy are emphasized in the lecture format during the morning session before practice on the mannequin.

#### 2.3.2. Hands-On Workshop

The first three stations are devoted to flexible fiberoptic intubation and include adult FFI, pediatric FFI, and FFI via laryngeal mask airway using the Aintree intubation catheter [6, 9, 10]. The pediatric difficult airway station incorporates pediatric video laryngoscopy using the pediatric video Mac laryngoscope [40]. Uses of rescue supraglottic airway devices, Combitube, intubating LMA (Fastrach LMA), and lightwand (Trachlight) are practiced at the supraglottic airway devices station [21–23]. The laryngeal tube (King LT-D supralaryngeal airway) has been added recently to this station [24]. The laryngeal tube is used in the out-of-hospital emergency airway management and can be replaced with an ETT by using the fiberoptic-guided placement of the Aintree intubation catheter through the laryngeal tube [25]. The video laryngoscopy station includes the three most often used video laryngoscopes: GlideScope, Storz C-MAC, and McGrath video laryngoscopes [12–20]. Residents are able to see and compare differences between the video laryngoscopes. Proper video laryngoscopy technique is taught in order to prevent airway injury and improve success of the advancement of the endotracheal tube through the glottic opening [14, 19, 20, 41]. One station is devoted to optically enhanced laryngoscopy. The Airtraq optical laryngoscope and Bonfils fiberoptic stylet are available for practice at this station [26–31]. Separation of the lungs during the difficult airway management is emphasized at the bronchial blockers station. Application of Arndt and Cohen bronchial blockers can be mastered [42]. A station is devoted to extubation of the difficult airway station where residents can become familiar with adult and pediatric airway exchange catheters and double lumen tube exchange catheter [32–34]. Two stations are devoted to the invasive airway: retrograde wire intubation and cricothyrotomy [38, 39]. Equipment for these two stations is supplied by Cook Medical.

## 3. Discussion

Although the Accreditation Council for Graduate Medical Education (ACGME) Program Requirements for Graduate Medical Education in Anesthesiology emphasizes that resident must obtain significant experience with a broad spectrum of airway management techniques (e.g., performance of fiberoptic intubation, double lumen endotracheal tube, endobronchial blockers) [43], there is no specific information on what constitutes the minimum clinical experience that should be obtained in order to ensure competency with the specific airway management technique. A rotation in advanced airway management should be part of the required curricula in anesthesiology residency programs. Standardization of resident training in advanced airway management can be achieved by a clinical certification process and established competencies for the training in advanced airway management techniques and devices. A survey concerning formal training in advanced airway management techniques was performed twice [3, 44]. In 1995, only 27% of the anesthesiology programs reported to have a formal advanced airway rotation in their curriculum [44]. The majority of these airway rotations (60%) were less than two weeks in duration. The other survey conducted in 2002 demonstrated that only 33% of the anesthesiology programs had a dedicated airway rotation [3]. Of these, more than 61% of the rotations were only one week in duration [3]. A well-designed survey would be valuable in assessing current practices within US anesthesiology residency programs.

We believe that a comprehensive airway management educational program is necessary to help anesthesiology residents earn “expert” status in difficult airway management. Three years ago, we instituted in our institution a formal educational program in advanced airway management for senior residents in anesthesiology which consist of a two-to-four-week clinical rotation together with a semiannual difficult airway workshop. Although offered as an elective rotation, all CA-3 residents have elected to do the rotation during the past three years. In a survey of departmental educational activities, both the AAT rotation and the Difficult Airway Workshop have been voted by the residents as the best educational activities for the past two consecutive years. We believe that this program has resulted in improved technical skills of our residents, and improved patient safety and patient care during the difficult airway management. Future studies should focus on determining if education in advanced airway management can result in decrease in airway related morbidity and mortality and overall better patients' outcome during difficult airway management.

## Appendix

### Difficult Airway Cart—Supply List

Top of Cart
  Monitor—Storz (Karl Storz Endoscopy)

Shelf 1
  Light source—Storz (Karl Storz Endoscopy)
  Video box—Storz (Karl Storz Endoscopy)

Shelf 1—Drawer 1
  Lube spray—Sklar Instruments, West Chester, PA, USA
  Glass atomizer—Sunrise Medical, Somerset, PA, USA

Shelf 1—Drawer 2
  Video MAC blade—Karl Storz, Germany
  C-MAC Video D-Blade—Karl Storz, Germany

Shelf 1—Drawer 3
  MAC 3 & 4 blades—Heine, Germany
  Adult handle—Heine, Germany
Drawer 1
  DCI camera head—Storz (Karl Storz Endoscopy)
  DCI video cable—Storz (Karl Storz Endoscopy)
  Fiberoptic video cable—Storz (Karl Storz Endoscopy)
Shelf 2
  Blue tote for contaminated flexible
  fiberoptic bronchoscope—Storz (Karl Storz Endoscopy)

Drawer 2
  LMA, sizes 3, 4, 5—Ambu, Inc., Glen Burnie, MD, USA
  Intubating LMA, sizes 3, 4, 5—LMA Fastrach (LMA North America, San Diego, CA, USA)
  Adult intubating stylet—three-Cardinal Health, Dublin, OH, USA
  Parker flex-tip endotracheal tubes, size 6.5, 7.0, 7.5, 8.0—Parker Medical, Englewood, CO, USA
Drawer 3
  Emergency transtracheal airway catheter—6.0 Fr/5 cm, Cook Medical, Bloomington, IN, USA
  Melker Emergency Cricothyrotomy Catheter set–Cook Medical, Bloomington, IN, USA
  Cook Retrograde intubation set—Cook Medical, Bloomington, IN, USA
  Light Wand lighted stylet and handle—Vital Signs Colorado, Inc., Englewood, CO, USA
  Endotracheal tube introducer, Sun Med, Largo, FL, USA

Drawer 4
  Combitube—Tyco Healthcare, Pleasanton, CA, USA
  Nebulizer set—three-Salter Labs, Arvin, CA, USA
  Jet ventilator tube—Medtronic Xomed, Inc., Jacksonville, FL, USA

Right of Cart, Bin 1
  Ovassapian airways—two-Teleflex Medical, Research Triangle Park, NC, USA
  Williams airways, size 9 & 10—two each
  Bite block/tongue depressor stick—Hudson Respiratory Care, Inc., Temecula, CA, USA

Right of Cart, Bin 2
  Double swivel connector with port—five—King System Corporation, Noblesville, IN, USA

Right of Cart, Bin 3
  Suction valves—four—Storz (Karl Storz Endoscopy)
  MADgic atomizer device—three—Wolfe Tory Medical, Inc., Salt Lake City, UT, USA

Right of Cart, Bin 4
  Nasal decongestant—Rhinall/Scherer Laboratories, Inc., Atlanta, GA, USA
  Cetacaine spray—Cetylite Industries, Inc., Pennsauken, NJ, USA

Right of Cart, Bin 5
  2% viscous lidocaine—Hi-Tech Pharmacal Co. Inc., Amityville, NY, USA
  4% lidocaine hydrochloride—topical solution—Roxane Laboratories, Inc., Columbus, OH, USA
  2% lidocaine jelly—Akron, Inc., Lake Forest, IL, USA
  5% lidocaine ointment—E. Fougera & Co., Melville, NY, USA

Right of Cart, Bin 6
  O_2 _supply tubing—three-Teleflex Medical, Research Triangle Park, NC, USA

Right of Cart, Bin 7
  Defogger—two-DeRoyal, Powell, TN, USA
  Alcohol preps—ten
  Difficult intubation stickers—five.
